# Antimicrobial Role of RNASET2 Protein During Innate Immune Response in the Medicinal Leech *Hirudo verbana*

**DOI:** 10.3389/fimmu.2020.00370

**Published:** 2020-03-06

**Authors:** Nicolò Baranzini, Annarosaria De Vito, Viviana Teresa Orlandi, Marcella Reguzzoni, Laura Monti, Magda de Eguileor, Elena Rosini, Loredano Pollegioni, Gianluca Tettamanti, Francesco Acquati, Annalisa Grimaldi

**Affiliations:** ^1^Department of Biotechnology and Life Science, University of Insubria, Varese, Italy; ^2^Department of Medicine and Surgery, University of Insubria, Varese, Italy

**Keywords:** RNASET2, antimicrobial activity, LTA, innate immunity, medicinal leech

## Abstract

The innate immune response represents a first-line defense against pathogen infection that has been widely conserved throughout evolution. Using the invertebrate *Hirudo verbana* (Annelida, Hirudinea) as an experimental model, we show here that the RNASET2 ribonuclease is directly involved in the immune response against Gram-positive bacteria. Injection of lipoteichoic acid (LTA), a key component of Gram-positive bacteria cell wall, into the leech body wall induced a massive migration of granulocytes and macrophages expressing TLR2 (the key receptor involved in the response to Gram-positive bacteria) toward the challenged/inoculated area. We hypothesized that the endogenous leech RNASET2 protein (*Hv*RNASET2) might be involved in the antimicrobial response, as already described for other vertebrate ribonucleases, such as RNase3 and RNase7. In support of our hypothesis, *Hv*RNASET2 was mainly localized in the granules of granulocytes, and its release in the extracellular matrix triggered the recruitment of macrophages toward the area stimulated with LTA. The activity of *Hv*RNASET2 was also evaluated on *Staphylococcus aureus* living cells by means of light, transmission, and scanning electron microscopy analysis. *Hv*RNASET2 injection triggered the formation of *S. aureus* clumps following a direct interaction with the bacterial cell wall, as demonstrated by immunogold assay. Taken together, our data support the notion that, during the early phase of leech immune response, granulocyte-released *Hv*RNASET2 triggers bacterial clumps formation and, at the same time, actively recruits phagocytic macrophages in order to elicit a rapid and effective eradication of the infecting microorganisms from inoculated area.

## Introduction

The RNase T2 family is represented by extracellular ribonucleolytic enzymes that act at an optimal pH of about 4.0–5.0 and have been found variously distributed throughout *taxa*, from viruses to higher eukaryotes (1). Strikingly, despite their highly conserved biochemical and structural features, T2 RNases from different organisms show a marked pleiotropic nature, being involved in an impressive range of biological functions, often related to stress response and host defense (1, 2).

Indeed, T2 RNases have acquired throughout evolution a range of biological functions, such as control of cell senescence (3), induction of oxidative stress-mediated apoptosis (4), cytotoxicity (5), regulation of cell motility/migration by cytoskeletal reassembly (6), modulation of angiogenesis (7), regulation of self-incompatibility in plants (8, 9), and tumor suppression (10). In addition, T2 RNases counteract harmful agents to protect germinal seeds from different types of plant pathogens, such as viruses or bacteria (11).

The pleiotropic roles of T2 RNases are further suggested by the observation that several biological processes regulated by members of this enzyme family do not depend on their catalytic activity (1). In keeping with such pleiotropic role, besides extracellular compartments, T2 RNases have been reported in cytoplasmic vacuoles, lysosomes, P-bodies, and mitochondria (6, 12–14), further arguing in support to their involvement in a wide range of key biological processes.

Recently, our group begun investigating the role of T2 RNases in the medicinal leech *Hirudo verbana*, in order to better define the host defense role of this class of proteins in an invertebrate model showing a very simple anatomy, coupled to a marked similarity with vertebrates concerning the cellular and molecular effectors involved in inflammatory processes (15). Noteworthy, we were able to confirm the pro-inflammatory role of T2 RNases in this invertebrate model, carried out by means of recruitment and activation of cells from the monocyte/macrophage lineage, as previously defined in mammalian experimental models (10). This finding clearly suggests that stimulation of innate immunity-mediated host defense represents a key evolutionarily conserved role for T2 RNases (16, 17).

Furthermore, we found experimental evidence in support of a putative role for T2 RNases in host defense against bacterial infections as well, since lipopolysaccharide (LPS) injection into the leech body wall triggered a marked increase in the expression levels of endogenous leech T2 RNase (named *Hv*RNASET2) protein in both host macrophages and granulocytes. Moreover, recombinant *Hv*RNASET2 was apparently able to affect bacterial cell integrity *in vivo* (16, 17).

Although the molecular mechanisms by which T2 RNase enzymes act in the antimicrobial process are still unknown, this ability is reminiscent to that previously described for some members of the RNase A superfamily, which, unlike T2 RNases, has been described only in vertebrates (18). For instance, the class A human RNase3 protein, also called eosinophil cationic protein (ECP) (19), acts as a strong eosinophil-mediated antimicrobial protein or peptide (AMPs) independently from its ribonucleolytic activity (20). ECP is released during eosinophil activation from the inner secondary cytoplasmatic granules to the extracellular environment and, after specific interaction with bacterial cells, it permeabilizes their external membranes in order to disrupt them (21–23). ECP is active against different types of bacteria (24) and shows a high affinity to LPS, a component of the outer membrane of Gram-negative bacteria. By binding to bacterial cell membranes and subsequently destabilizing them, ECP shows a carpet-like anti-bacterial mechanism that recalls many host defense antimicrobial proteins or peptides (20). In addition, its N-terminal region induces the formation of bacterial clumps, thus promoting a systematic elimination by immune cells (25).

Other class A ribonucleases, such as RNase 2 and RNase 7, act as “alarmins,” molecules passively released by necrotic cells or actively secreted by immune or epithelial cells in order to signal to the innate and adaptive immune system the occurrence of a wide range of dangerous events, such as those occurring during pathogen infection or tumor progression. As such, alarmins promote inflammatory responses, usually mediated by Toll-like receptor family members (TLRs) (26, 27). Among TLRs, TLR2, and TLR4 represent the most significant group of PRRs (pattern recognition receptors), which are evolutionary conserved both in vertebrate and in invertebrate species (28–30) and are expressed by immune cell membranes. These receptors mediate the recognition of conserved biomolecules known as pathogen-associated molecular patterns (PAMPs), such as lipoteichoic acid (LTA) and LPS, which are normally displayed in the external membrane of Gram-positive and Gram-negative bacteria, respectively. Noteworthy, human RNASET2 has also been suggested to act as an alarmin, being actively released under a wide range of stressful conditions (4, 6, 31).

Starting from these assumptions, we adopted *H. verbana* as an experimental model to gain more insights into the role of *Hv*RNASET2 as a host defense protein and confirmed its antimicrobial action against Gram-positive bacteria in both *in vivo* and *in vitro* experimental settings.

## Materials and Methods

### *H. verbana* Recombinant r*Hv*RNASET2: Cloning and Expression

The mRNA sequence of *H. verbana* RNASET2 was obtained from an *in silico* leech transcriptome database (http://genomes.sdsc.edu/leechmaster/database/) (32): the >EN-124k-90-group2043 coding the full sequence was selected. In parallel, mRNA extraction from the leech body wall was performed and the sample was treated with DNase (Turbo DNA-*free*^TM^ Kit, Invitrogen) to remove all DNA traces. After reverse transcription with oligo dT (High-Capacity cDNA Reverse Transcription Kit, Applied Biosystems™), different couples of primers were used, resulting in a partial amplification of the coding sequence. Therefore, an overlap extension PCR was performed to obtain the full coding sequence of *Hv*RNASET2. The primers used in the first two PCR amplifications were as follows:

First sample: Fw: 5′-CGTAGAATTCAAGTAATTAAATCTGATTCGGAGTG-3′ (fw1-e); Rev: 5′-ATATTGCAGTGGTTCATTACGTGGA-3′.

Second sample: Fw: 5′-TCCACGTAATGAACCACTGCAATAT-3′; Rev: 5′-CGTAGAATTCTGTGTAAAAGGGAATATTAGATCAAG-3′ (rev7-e).

Both the products were used as template in a third PCR performed using the external primers (fw1-e + rev7-e—the underlined bases represent restriction site for EcoRI). After digestion with EcoRI, the product was cloned in pBluescript.

Subsequently, the leech *Hv*RNASET2 coding sequence (BankIt2095553 Hirudo MH325331) was amplified without the predicted signal for secretion, using the following primers:

5′-CGTAGAATTCAGGCCTCTGAAGGAAGAATT-3′; 5′-CGTATCTAGACCTGAGTTTGAATGAATTTGGTT-3′ as forward and reverse primer, respectively (the underlined sequences represent restriction sites for EcoRI and XbaI). After digestion with EcoRI and XbaI, the product was cloned into the pPICZαA expression vector for heterologous expression in the yeast *Pichia pastoris*, as previously described (33).

To introduce a 6XHIS tag at the N-terminus of the protein, a useful sequence for protein purification, the pPICZαA-*Hv*RNASET2 construct was digested at EcoRI site. The tag was introduced by amplification using the following primer pair (as an insert):

5′-AATTCCATCACCACCATCATCACG-3′; 5′-AATTCGTGATGATGGTGGTGATGG-3′.

Recombinant DNA was purified from several clones and, after control sequencing (BMR, Padova, Italy), the pPICZαA-*Hv*RNASET2 expression vector was used to transform the X33 *P. pastoris* strain. Briefly, the RNASET2-coding construct was linearized within the 3'AOX region with PmeI restriction enzyme and transformed into yeast with the lithium chloride method (34). The methanol utilization test was carried out as a control to verify the correct yeast phenotype.

Subsequently, a selected clone was inoculated into 50 ml of BMGY medium (BMGY/BMMY: 1% yeast extract, 2% bactopeptone, 1.34% yeast nitrogen base, 4 × 10^−5^% biotin and either 1% glycerol or 0.5% methanol). Cultures were grown at 30°C overnight until they reached an optical density at 600 nm (OD_600_) between 2 and 6. After centrifugation, cell pellets were resuspended in 1.3 L BMMY at a starting OD_600_ of 1 for induction of protein expression. Every day (for 7 days), fresh methanol (with a final concentration of 0.5%) was added to cultures.

### Purification of the Recombinant r*Hv*RNASET2

The supernatant was concentrated by ultrafiltration using an Amicon stirred cell (Merck Millipore) equipped with a 10-kDa membrane to a final volume of 40 ml and extensively dialyzed against 20 mM sodium phosphate and 150 mM NaCl, pH 7.5. The sample was added with NaCl up to 1 M final concentration and the protein was purified using a HiTrap chelating affinity column (5 ml) (GE Healthcare) previously loaded with 100 mM NiCl_2_ and equilibrated with 20 mM sodium phosphate, 1 M NaCl, pH 7.5. The column was washed with this buffer until the absorbance value at 280 nm was that of the buffer. r*Hv*RNASET2 was eluted with the same buffer added with 100 mM imidazole; the fractions were equilibrated with 20 mM sodium phosphate and 150 mM NaCl, pH 7.5, by gel-permeation chromatography (PD10 column, GE Healthcare). The amount of protein was determined by using the absorbance intensity at 280 nm and a molar extinction coefficient of 66 mM^−1^ cm^−1^ (6). The recombinant r*Hv*RNASET2 was isolated as a single band at ≈36 kDa with >90% purity as judged by SDS-PAGE analysis: ≈3.5 mg of purified enzyme per liter of fermentation broth was obtained.

### Endotoxin Removal

Endotoxins were removed from the purified protein according to the procedure reported in (35). Briefly, the protein sample was added with 1% Triton X-114, incubated at 4°C for 30 min and then at 37°C for 10 min, and finally centrifuged at 16,000 *g* for 15 min, at room temperature. The supernatant was recovered, and all the steps were repeated two times, plus a final step without Triton X-114. The removal of endotoxins was assessed with a LAL test (PYROGENT^TM^ Gel Clot LAL Assay, LONZA).

### Animals and Treatments

Adult leeches (*H. verbana*, Annelida, Hirudinea, from Ricarimpex, Eysines, France), measuring 10 cm, were kept in lightly salted water (NaCl 1.5 g/ml) in aerated tanks at 19–20°C. Before injection and/or dissection, leeches were anesthetized with a 10% ethanol solution and all treatments were performed at the 80th superficial metamere from the oral sucker. Animals were randomly split into separate experimental groups (three individuals for each time point) and submitted to various protocols and treatments, as described below:

Group 1: injection with 100 μl of sterilized phosphate buffer saline (PBS; 138 mM NaCl, 2.7 mM KCl 4.3 mM Na2HPO4, and 1.5 mM KH2PO4, pH 7.4) followed by evaluation at 30-min, 1-h, 3, 6, and 24-h time points, to confirm that PBS alone does not induce an immune response.
Group 2: injection with 100 μl of PBS containing 100 ng/ml of LTA from *Bacillus subtilis* (Sigma Aldrich, St. Louis, MO, USA) followed by evaluation at 30-min, 1-h, 3, 6, and 24-h time points, to stimulate an inflammatory response and to evaluate the expression of TLR2 and TNF-α in cells involved in the immune response. The optimal LTA concentration required to induce significant cell migration in leeches was determined based on our previous work (36).
Group 3: injection with 100 μl of PBS containing 1 μg/ml of CyP, an LPS-like molecule extracted from the cyanobacterium *Oscillatoria Planktothrix* FP1 [cyanobacterial product (CyP)] that acts as a potent and selective antagonist of bacterial LPS (30, 37) followed by evaluation at 30-min, 1-h, 3, 6, and 24-h time points. This treatment was performed to exclude any possible interaction between LTA and the LPS receptor TLR4 (30).
Group 4: injection with 100 μl of PBS containing 1 μg/ml of CyP plus 100 ng/ml of LTA obtained from *B. subtilis* (Sigma-Aldrich) followed by evaluation at 30-min, 1-h, 3, 6, and 24-h time points. This treatment was performed to confirm the TLR2 specificity in LTA recognition and to exclude any possible interaction between LTA and TLR4 (30).
Group 5: injection of 100 μl of sterile PBS containing 1 μg of rabbit polyclonal anti-TLR2 antibody (Abcam, Cambridge, UK, ab-213676) followed, after 30 min, by LTA injection to perform antibody-mediated neutralization as a specificity control to functionally block LTA recognition by TLR2. The samples were analyzed 6 h after LTA treatment.
Group 6: injection with 100 μl of PBS containing methicillin-susceptible *Staphylococcus aureus* ATCC 6538P (10^7^ CFU/ml) followed by evaluation at 3 h.
Group 7: injection with 100 μl of a PBS solution containing *S. aureus* ATCC 6538P (10^7^ CFU/ml) and 10 μM recombinant *H. verbana* RNASET2 protein (*Hv*RNASET2, accession number BankIt2095553 Hirudo MH325331) followed by evaluation at 3 h.
Group 8: injection with 100 μl of a PBS solution containing *S. aureus* ATCC 6538P (10^7^ CFU/ml) and 10 μM r*Hv*RNASET2 pre-treated with 1 μg of an anti-RNASET2 antibody followed by evaluation at 3 h, as a specificity control to functionally block *Hv*RNASET2 activity (30).
Group 9: animals injected with 300 μl of liquid MG supplemented with 50 μl of *S. aureus* pre-treated with 50 ng of r*Hv*RNASET2, used to selectively isolate macrophages recruited by *Hv*RNASET2 (16) and to evaluate their enhanced phagocytic activity, were analyzed at T7 days.
Group 10: animals injected with 300 μl of liquid MG supplemented with 50 μl of *S. aureus* pre-treated with 50 ng of r*Hv*RNASET2 and 1 μg of the polyclonal antibody RNASET2, as a specificity control to functionally block *Hv*RNASET2 activity, and analyzed at T7 days.

### Light and Electron Microscopy

Samples, dissected from the area of injection in each experimental group, were fixed for 2 h in 0.1 M cacodylate buffer at pH 7.4 containing 2% glutaraldehyde. After several washes in the same buffer, tissue samples were postfixed for 1 h with 1% osmium tetroxide in cacodylate buffer, pH 7.4, and subsequently embedded in an Epon-Araldite 812 mixture (Sigma-Aldrich, Milan, Italy), after serial ethanol dehydration (70, 90, 100%). Tissue sections were obtained with a Reichert Ultracut S ultratome (Leica, Wien, Austria). Semi-thin sections (0.7 μm) were stained by conventional methods, using crystal violet and basic fuchsin [according to Moore et al. (38)], and observed under a light microscope Nikon Eclipse Ni (Nikon, Tokyo, Japan). Data were recorded with a DS-5M-L1 digital camera system (Nikon). Ultrathin sections (80 nm) were collected on copper grids (300 mesh, Sigma-Aldrich, Milan, Italy), counterstained by uranyl acetate and lead citrate, and observed with a Jeol 1010 EX transmission electron microscope TEM (Jeol, Tokyo, Japan). Data were recorded with a MORADA digital camera system (Olympus, Tokyo, Japan).

*S. aureus* ATCC 6538P cells were grown overnight in Müller Hinton broth 2 (MHB2, 0.3% beef infusion solids, 1.75% casein hydrolysate, and 0.15% starch) with continuous shaking at 200 rpm at 37°C and then transferred to fresh medium to reach the exponential growth phase. Subsequently, bacteria were suspended in PBS with the recombinant enzyme r*Hv*RNASET2 (10 μM) for 3 h at 20°C, and then centrifuged for 10 min at 12,000 rpm. After supernatant removal, bacterial pellets were fixed with Karnovsky fixative (2% paraformaldehyde and 2.5% glutaraldehyde in 0.1 M cacodylate buffer, pH 7.2) for 1 h at 4°C and then processed for TEM microscopy as above described.

3D imaging was obtained by scanning electron microscopy (SEM). After 3 h from r*Hv*RNASET2 (10 μM) treatment, bacteria were fixed in Karnovsky fixative for 30 min, washed in 0.1 M cacodylate buffer (pH 7.2), and post-fixed in a solution of 1% osmium tetroxide and potassium ferrocyanide for 1 h. After several washes in PBS (pH 7.2) and dehydration with an increasing scale of ethanol, 20 μl of bacterial pellet resuspended in ethanol 100% was dried onto glass slides and finally subjected to critical point drying with hexamethyldisilazane. Images were acquired using the SEM-FEG XL-30 microscope (Philips, Eindhoven, The Netherlands).

### Immunogold Staining at TEM

Samples were fixed for 2 h at 4°C with 4% paraformaldehyde and 0.5% glutaraldehyde in PBS, dehydrated in ethanol series, and embedded in an Epon-Araldite 812 mixture (Sigma-Aldrich). Ultrathin sections, obtained as above, were collected on gold grids (300 mesh, Sigma-Aldrich). After etching with 3% NaOH in absolute ethanol (39), they were incubated for 30 min in blocking solution containing PBS, 1% bovine serum albumin (BSA), and 0.1% Tween and then with the polyclonal primary antibody rabbit anti-human RNASET2 (40) diluted at 1:20 in blocking solution. After several washings with PBS, the primary antibody was visualized by immunostaining with the secondary goat anti-rabbit IgG (H+L)-gold conjugate antibody (GE Healthcare, Amersham, UK; particle size, 10 nm) diluted at 1:100 in blocking solution for 1 h. In control experiments, the primary antibody was omitted and sections were treated with BSA containing PBS and incubated only with the secondary antibodies. Sections were counterstained with uranyl acetate in water and observed at TEM, and data were recorded with a digital camera system as previously described.

### Immunofluorescence Assays

Samples, dissected from differently treated leech body wall, were embedded in Polyfreeze tissue freezing medium (OCT, Polysciences, Eppelheim, Germany), immediately frozen in liquid nitrogen. Cryosections (7 μm) from *S. aureus*-injected leeches were obtained with a cryotome (Leica CM1850), collected on gelatinous slides and counterstained with crystal violet and basic fuchsin for morphological analysis or with 0.1 mg/ml 4,6-diamidino-2-phenylinedole (DAPI, excitation and emission filter 360/420 nm) diluted 1:5000 in PBS to highlight bacterial DNA.

For immunofluorescence assays, slices were incubated for 30 min in blocking solution and then for 1 h at 37°C with the following polyclonal primary antibodies diluted in the same blocking solution: rabbit anti-RNASET2 (40), expressed by macrophages and granulocytes of leech (17) diluted 1:200; goat anti-CD11b (Santa Cruz Biotechnology, CA, USA, sc-28664) that specifically stains leech granulocytes (41) diluted 1:100; rabbit anti-*Hm*AIF-1 (kindly donated by Prof. Jacopo Vizioli, University of Lille 1, France), reacting with leech macrophages (36, 42), diluted 1:1000; rabbit anti-TNF-α (Abcam, Cambridge, UK, ab6671) diluted 1:200 reacting with leech homologous protein (30); and rabbit anti-TLR2 (Abcam, Cambridge, UK, ab213676, recognizing an epitope corresponding to amino acids 730–780 mapping to an internal region of TLR2 of human origin) diluted 1:200. After washing in PBS, samples were incubated for 45 min at room temperature, respectively with an anti-goat or anti-rabbit Cy5-conjugated (Jackson ImmunoResearch Laboratories, West Grove, USA) secondary antibodies (excitation filter 650 nm, emission filter 672 nm) diluted 1:250 in blocking solution. Double-labeling experiments that detect cells co-expressing CD11b/TLR2 or CD11b/RNASET2 were performed combining the following polyclonal primary antibodies: goat anti-CD11b and rabbit anti-TLR2 or goat anti-CD11b and rabbit anti-RNASET2. After washing in PBS, sections were incubated with a mix of the appropriate secondary antibodies: donkey anti-goat fluorescein isocyanate (FITC) conjugated (excitation filter 493 nm, emission filter 518 nm) and goat anti-rabbit Cy5 conjugated (Jackson ImmunoResearch Laboratories), diluted 1:200. To detect MyD88/TLR2, *Hm*AIF-1/TLR2, and *Hm*AIF-1/RNASET2 co-expressing cells, since the primary antibodies were raised in the same species, the method previously described was used (16). The primary antibodies rabbit anti-MyD88 or rabbit anti *Hm*AIF-1 were applied first, and then sections were incubated with the secondary antibody goat anti-rabbit (FITC) conjugated. Before the second staining cycle, sections were treated with rabbit IgG (Jackson ImmunoResearch Laboratories) diluted 1:25 for 2 h (43) and incubated with rabbit anti-TLR2 or anti-RNASET2. Subsequently, the sections were treated with the secondary goat anti-rabbit (Cy5) conjugated diluted 1:200. Tissue autofluorescence was reduced by treating sections with 1 mM CuSO_4_ in 50 nM ammonium acetate buffer (pH 5.0) for 15 min (44). In all sections, nuclei were counterstained for 5 min with DAPI. The primary antibodies were omitted in the negative control experiments and sections were incubated only with the secondary antibodies.

All samples were mounted with Cityfluor (Cityfluor Ltd, UK) and examined with a Nikon Eclipse Ni (Nikon, Tokyo, Japan) light and fluorescence microscope. Data were recorded with a Nikon digital sight DS-SM (Nikon) and combined with Adobe Photoshop (Adobe Systems, San Jose, CA, USA).

### Acid Phosphatase Reaction (ACP)

Samples taken from injected areas were embedded in OCT and frozen in liquid nitrogen. Cryosections (7 μm) were rehydrated with PBS for 5 min and stained as previously described (16).

### Western Blot Analysis

Leech tissues, dissected from the LTA-challenged areas, were promptly frozen in cryovials and homogenized with a mortar. Homogenates (10 μl per milligram of tissue) were suspended in RIPA buffer (150 mM NaCl, 1% NP-40, 0.5% sodium deoxycholate, 0.1% SDS, and 50 mM Tris–HCl, pH 8.0) in the presence of protease and phosphatase inhibitors and kept overnight on a rotation mixer at 4°C. The particulate was removed by centrifugation at 13,000 rpm for 20 min at 4°C in a refrigerated Eppendorf Minispin microcentrifuge (Hamburg, Germany). After denaturation at 95°C for 5 min, protein concentrations were assayed with Coomassie Brilliant Blue G-250 protein assay (Pierce, Rockford, IL, USA) and BSA was used as standard. Ten microliters of denatured proteins (2 mg/ml final concentration) was loaded on gel 12% acrylamide minigels for SDS-PAGE analyses. The SDS-PAGE separated proteins were transferred onto a nitrocellulose filter by means of a gel transfer system by applying 350 mA for 2 h. After pre-incubation for 2 h in continuous stirring with a blocking solution containing 5% milk in Tris buffered saline (TBS: 50 mM Tris–HCl, pH 7.5, and 150 mM NaCl), membranes were treated overnight at 4°C with the following primary rabbit polyclonal antibodies: anti-RNASET2 (40), anti-TLR2 antibody (Sigma-Aldrich), and anti-TNF-α (Abcam) diluted 1:250 in blocking solution. After several washes with TBST (0.1% Tween 20 in TBS), nitrocellulose membranes were incubated with a secondary anti-rabbit IgG antibody horseradish peroxidase conjugated (Jackson ImmunoResearch Laboratories) diluted 1:7500 in blocking solution for 1 h at room temperature. To reveal the immunocomplexes, the membranes were incubated with luminol LiteAblot® PLUS Enhanced Chemiluminescent Substrate (EuroClone S.p.A., Pero, Italy) and exposed to a Kodak X-Omat AR film. Subsequently, nitrocellulose membranes were placed in stripping solution [62.5 mM Tris-HCl, pH 6.7, 2% (w/v) SDS, and 100 mM 2-mercaptoethanol] for 30 min at 50°C, washed in TBS, and incubated with blocking solution for 30 min and then with a rabbit anti-human polyclonal antibody IgG recognizing the housekeeping protein D-glyceraldehyde-3-phosphate dehydrogenase (GAPDH) diluted 1:1000 (Proteintech, Chicago, USA). Immunolabeled bands were detected using an anti-rabbit secondary antibody peroxidase conjugated (Jackson ImmunoResearch Laboratories) diluted 1:7500 in blocking solution for 1 h at room temperature. The processed blots, before and after stripping, were scanned and, for quantification analysis, were subjected to densitometry analysis using ImageJ software package (http://rsbweb.nih.gov/ij/download.html). The recorded intensities of the GAPDH bands were used as an internal control to correct for differences in the samples loading on the gels and the bands were normalized with GAPDH using the ImageJ software package. The expression levels of *Hv*RNASET2, TLR2, and TNF-α were reported relatively to control PBS-injected animals.

### Bacterial Viability Assay

Viable counts (expressed as colony-forming units per milliliter, CFU/ml) were estimated by employing the plate count technique: a volume (0.1 or 0.01 ml) of undiluted or serially diluted samples was plated on nutrient agar plates and incubated for 24 h at 37°C to evaluate the viable cells.

In order to evaluate the r*Hv*RNASET2 minimal inhibitory concentration (MIC) toward *S. aureus* ATCC 6538P, according to guidelines from CLSI (Clinical and Laboratory Standard Institute; Methods for Dilution Antimicrobial Susceptibility Tests for Bacteria That Grow Aerobically; Approved Standard, 10th ed. Wayne, PA: Clinical and Laboratory Standards Institute; 2015CLSI document M07-A10), bacteria were grown overnight in Tryptic Soy Broth at 37°C and diluted 200-fold to give a cellular concentration of 10^6^ c.f.u. ml/1. Two-fold dilutions of r*Hv*RNASET2 (from 10 to 0.039 μM) were added to bacterial samples. The dilution series were then tested for their effect on microbial growth after 24 h of incubation at 37°C, and MIC values were defined as the minimal concentration of r*Hv*RNASET2 at which no turbidity could be detected.

### Statistical Analysis

Western blot and immunofluorescent experiments were performed in triplicate and data represent the mean values ± SD. The percentages of CD11b^+^ and *Hm*AIF-1^+^ cells were assessed by analyzing five different slides (random fields of 45,000 μm^2^ for each slide) for each experimental time point using the ImageJ software package. Cells in the chosen fields were counted by hand as granulocytes if they were CD11b^+^ Cy5 labeled or as macrophages if they were *Hm*AIF-1^+^ Cy5 labeled. Statistical analyses were performed using GraphPad Prism 7 (GraphPad Software, La Jolla, CA, USA), and differences were calculated by one-way ANOVA followed by Fisher's *post-hoc* test and *p* <0.05 was considered statistically significant.

## Results

Based on our previous results, showing that *Hv*RNASET2 was both detected in leech granulocytes and able to actively recruit macrophages in a bacterially infected area (17), we investigated the *Hv*RNASET2 antimicrobial action toward Gram-positive bacteria. To this aim, *in vivo* experiments were first performed to characterize the leech immune cells involved in the inflammatory response induced by LTA injection, a key component of the bacterial cell wall used to simulate a Gram-positive bacterial infection. Subsequently, the direct antimicrobial effect of the recombinant r*Hv*RNASET2 was evaluated by *in vitro* assays.

## Morphological Characterization of Immune Cell Types Involved in the Inflammatory Response Induced in Leeches by LTA Injection

**Figure 1:**
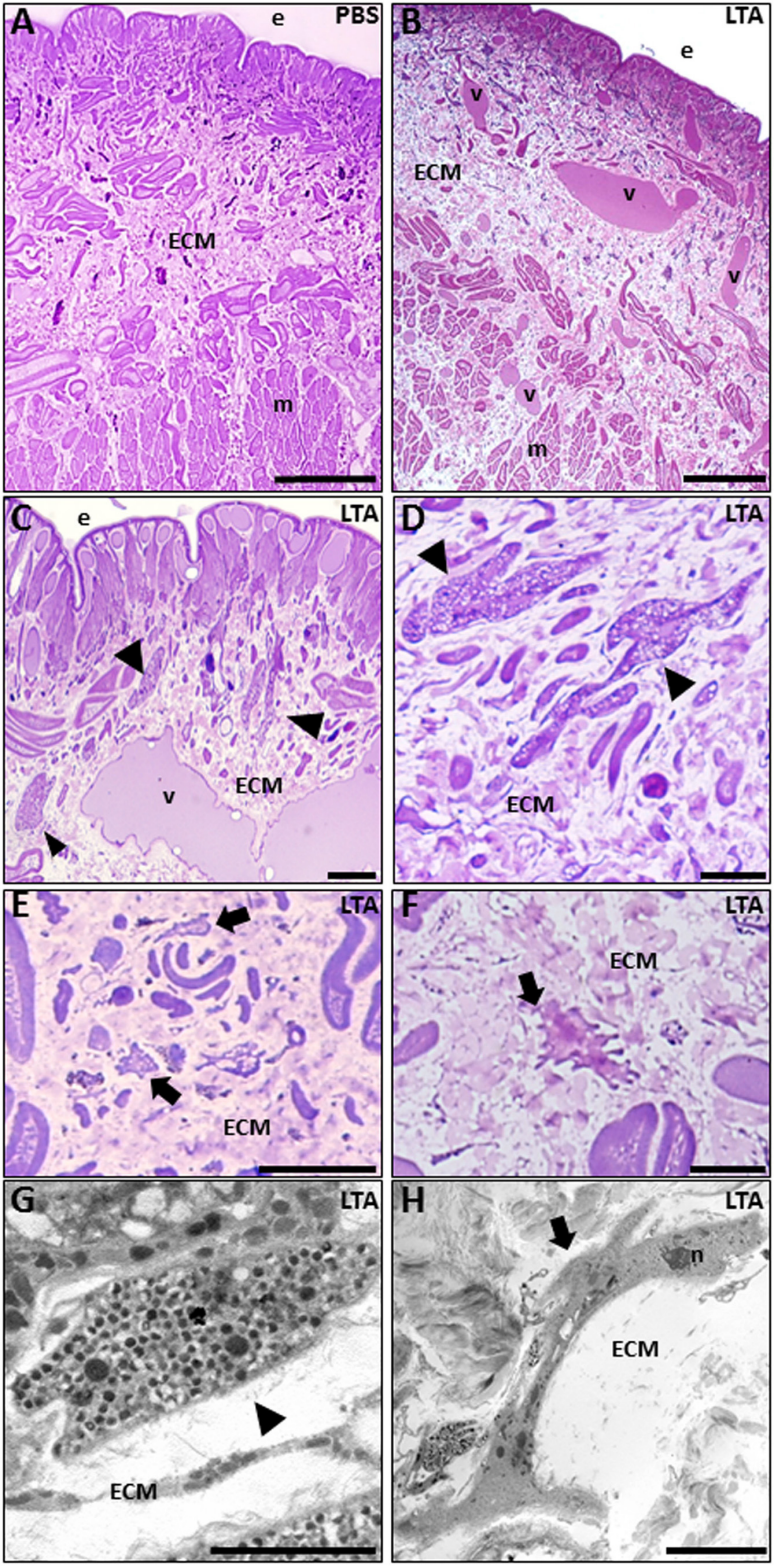
Images of morphological analyses of cross-sectioned leech body wall at light **(A–F)** and TEM microscopes **(G,H)**. In PBS-injected leeches **(A)**, tissue appears essentially avascular with few resident cells underneath the epithelium (e) and in the extracellular matrix (ECM) surrounding the muscle fibers (m). After LTA treatment **(B–D)**, new vessels (v), numerous granulocytes (arrowheads in **C,D**), and macrophages (arrows in **E,F**) are clearly visible among muscles and underneath the epithelium. TEM details show granulocytes (arrowhead in **G**), with dark granules inside the cytoplasm, and macrophages (arrow in **H**) characterized by the presence of pseudopodia. Bars in **(A–C)**: 100 μm; bars in **(D,F)**: 10 μm; bar in **(E)**: 50 μm; bar in **(G)**: 2 μm; bar in **(H)**: 5 μm. n, nuclei.

By means of light microscope analysis, the cross-sectioned body wall of PBS-injected leeches (Figure 1A) showed a typical cutaneous muscle sac, formed by well-defined epithelial and avascular muscular layers, where muscle fibers were arranged in distinct groups surrounded by a scant extracellular matrix (ECM). Among muscle fields, only a few resident immunocompetent cells were detectable.

By contrast, starting from 30 min following LTA injection, newly formed vessels and many infiltrating immune cells were clearly observable underneath the epithelium and in the ECM surrounding the muscle fibers (Figures 1B–H). In particular, several granulocytes were clearly recognizable both by light (Figure 1D) and TEM microscope analysis (Figure 1G) (17, 30). At 6 h post-injection, a high number of macrophages infiltrating the injected area (characterized by a ruffled surface due to the presence of pseudopodia, a typical feature of migrating cells) were readily detected as well (Figures 1E,F,H). LTA injection thus triggered a typical antibacterial response in leeches.

## CD11b and TLR2 Expression

**Figure 2:**
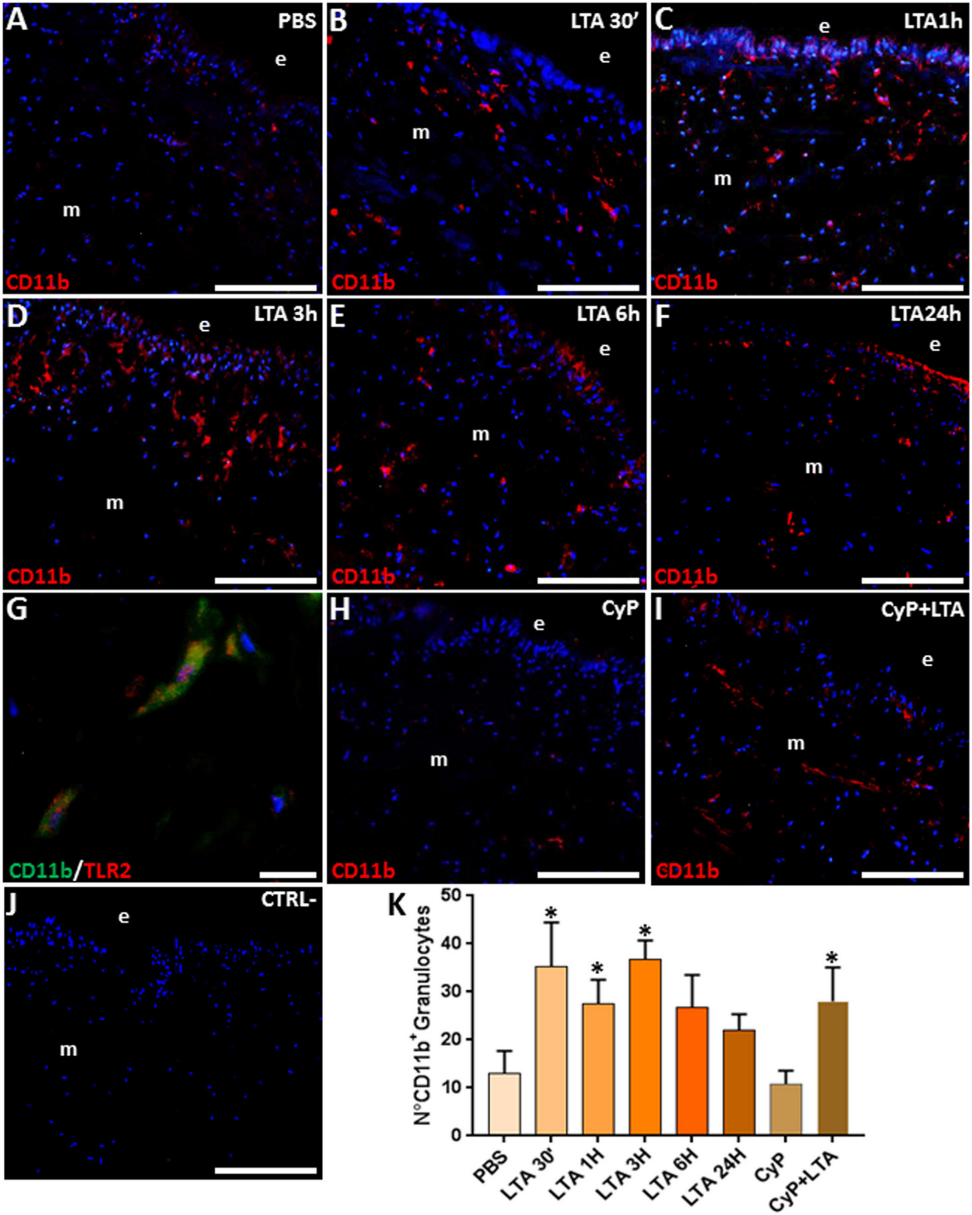
Immunofluorescence analyses on cryosections from leech body wall injected with PBS **(A)**, LTA **(B–G)**, CyP **(H)**, and CyP + LTA **(I)**. In PBS **(A)** and CyP **(H)** treated leeches, few CD11b^+^ granulocytes are detectable under the epithelium (e) and in the ECM surrounding muscles (m). The signal considerably increases after 30 min **(B)**, reaches a peak after 1 and 3 h **(C,D)**, and decreases after 6 h **(E)** and 24 h from treatment **(F)**. In CyP/LTA samples **(I)**, signal appears to be similar to LTA-challenged leeches. In negative control experiments **(J)**, where the primary antibody is omitted, no positive cells are detected. Double immunofluorescence assays **(G)**, using anti CD11b (green) coupled to TLR2 antibody (red), reveal that granulocytes express TLR2. The cell count is obtained on CD11b fluorescence signal for each treatment **(K)**. Cell nuclei were stained in blue by treatment with DAPI. Bars in **(A–F,H–J)**: 100 μm; bar in **(G)**: 10 μm. *p < 0.05.

Since these morphological observations correlated well with our previous finding following Gram-negative bacterial infection in leeches, which showed the massive recruitment of CD11b^+^ granulocytes expressing the TLR4-specific LPS receptor toward the challenged area (30), we focused on the immunophenotype of leech granulocytes activated in response to LTA injection. As expected, in control/PBS-injected leeches (Figure 2A), a low CD11b signal was detectable, indicating that the mechanical stress induced by the injection or the vehicle solution alone did not trigger a significant inflammatory effect.

By contrast, starting from 30 min up to 3 h following LTA injection, an increased number of CD11b^+^ granulocytes was clearly visible underneath the epithelium and crossing the ECM surrounding the muscle fibers (Figures 2B–D). The number of granulocytes decreased after 6 h (Figure 2E) and was drastically reduced 24 h post-treatment (Figure 2F). Double immunofluorescence assays coupling anti-CD11b and anti-TLR2 antibodies showed that these two markers were co-expressed in the granulocytes (Figure 2G), suggesting that the TLR2 receptor might be involved in Gram-positive bacteria recognition during the early phase of immune response in leeches. To confirm that the recognition of LTA did not involve the TLR4 pathways, which is specific for Gram-negative bacteria recognition, further analyses were performed by injecting the cyanobacterium selective TLR4 antagonist CyP (29, 30). In detail, leeches were injected with CyP (as a control) or with CyP added to LTA. Our data showed that the immune response caused by LTA treatment was not affected by Cyp (Figure 2H) and CD11b expression in LTA/Cyp-stimulated leeches was comparable to that found in tissues of leeches injected with LTA only (Figure 2I). These data suggest that CyP is closely connected to LPS-TLR4 interaction in leech and does not interfere with LTA-TLR2-specific signal transduction pathway. In control experiments, where the primary antibody was omitted, no signals were detected (Figure 2J). Furthermore, as demonstrated by cell counting performed on five representative images of each time lapse, the number of CD11b^+^ granulocytes changed during the different phases of inflammation and its increase was statistically significant in the earliest LTA- and CyP/LTA-induced inflammatory phase (Figure 2K).

**Figure 3:**
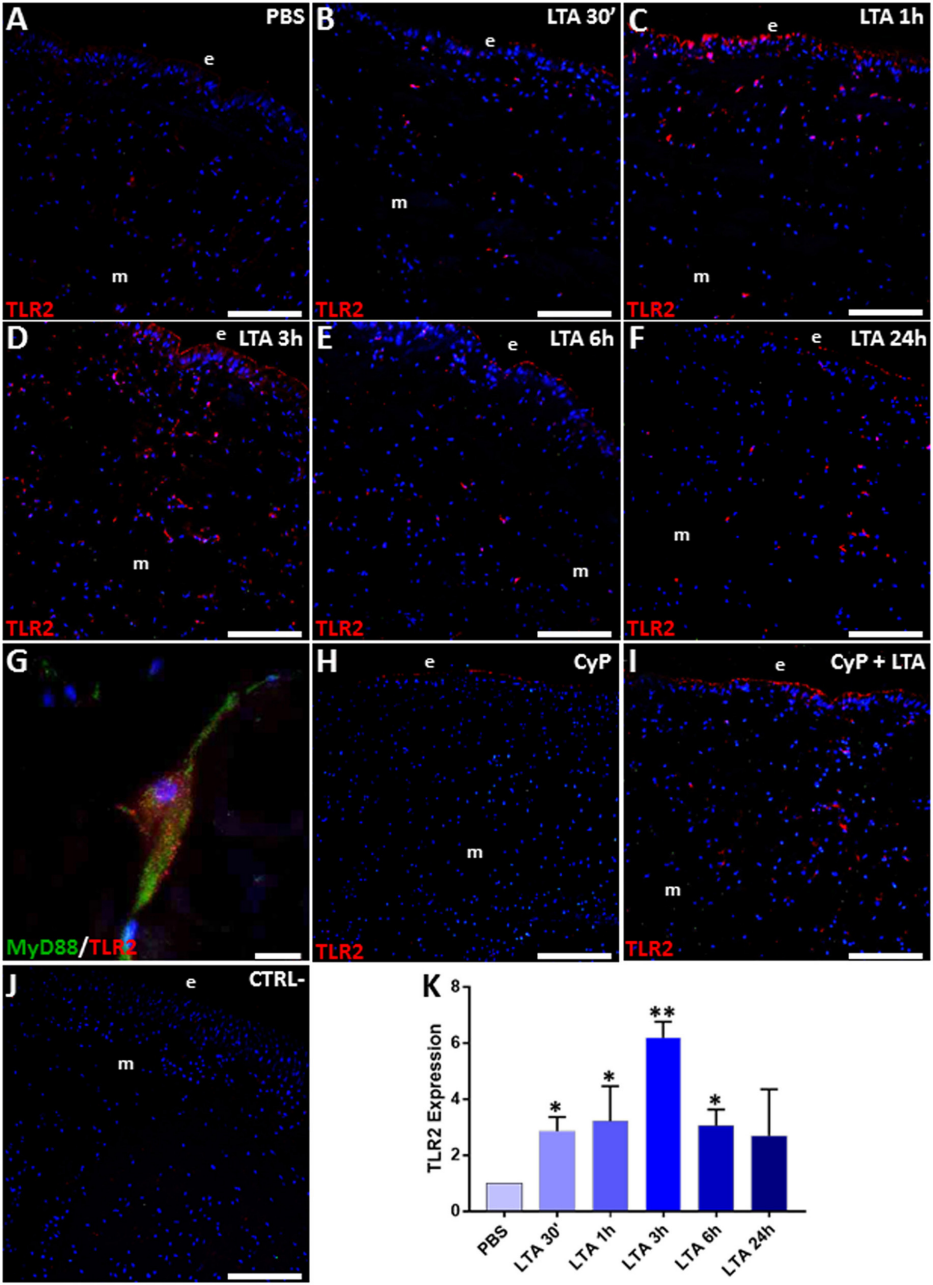
Immunofluorescence assays on cryosections from leeches injected with PBS **(A)**, LTA **(B–G)**, CyP **(H)**, and CyP+LTA **(I)**. In PBS**-**injected leech **(A)**, few TLR2^+^ cells were visible, whereas an increasing number of migrating immune-responsive cells located under the epithelium (e) and among muscles (m) are detected starting from 30 min until 3 h following LTA injection **(B–D)**. The signal turned out to decrease at 6 and 24 h post-treatment **(E,F)**. In CyP **(H)** and Cyp/LTA samples **(I)**, the number of TLR2^+^ cells appears to be similar to that of PBS and of LTA-challenged leeches, respectively. No positive cells are detected in negative control experiments **(J)**. Detail of double immunofluorescence stained with anti-TLR2 (in red) and MyD88 (in green) **(G)**. Cell nuclei are stained in blue with DAPI. The graph, relative to the Western blot analysis (see Figure S1) showing the TLR2 expression profile **(K)**. Bars in **(A–F,H–J)**: 100 μm; bar in **(G)**: 10 μm. *p < 0.05, **p < 0.001.

The expression of TLR2 and its downstream signaling pathway components was then evaluated by means of immunofluorescence and Western blot analyses. In PBS-injected control leeches (Figure 3A), a low fluorescent signal for TLR2 was detected. By contrast, an increased number of TLR2^+^ cells was detected 30 min after LTA injection, reaching a peak after 3 h (Figures 3B–D) to later decrease at 24 h post-treatment (Figures 3E,F). Moreover, double immunofluorescent assays showed a marked co-localization of MyD88 and TLR2 in the same cells (Figure 3G), thus confirming a MyD88-dependent activation pathway via TLR2 (45) in leech. Further studies using the CyP agonist confirmed that LTA recognition involved only TLR2 and its signaling pathway, while TLR4 was excluded. Indeed, a TLR2 signal comparable to that of control and of 3-h LTA-injected leeches was detected in both CyP- and Cyp/LTA-treated leeches, respectively (Figures 3H,I). No signal was detected in the negative control experiments in which tissue sections were incubated only with the secondary antibody (Figure 3J).

The increased expression level of TLR2 in LTA-injected leeches was also confirmed by immunoblot assays, showing the presence of a 109-kDa band, corresponding to the expected molecular weight of vertebrate TLR2 (Figure S1). As shown in the figure, TLR2 expression was highly increased in LTA-treated samples when compared to PBS-treated controls (Figure 3K).

**Figure 4:**
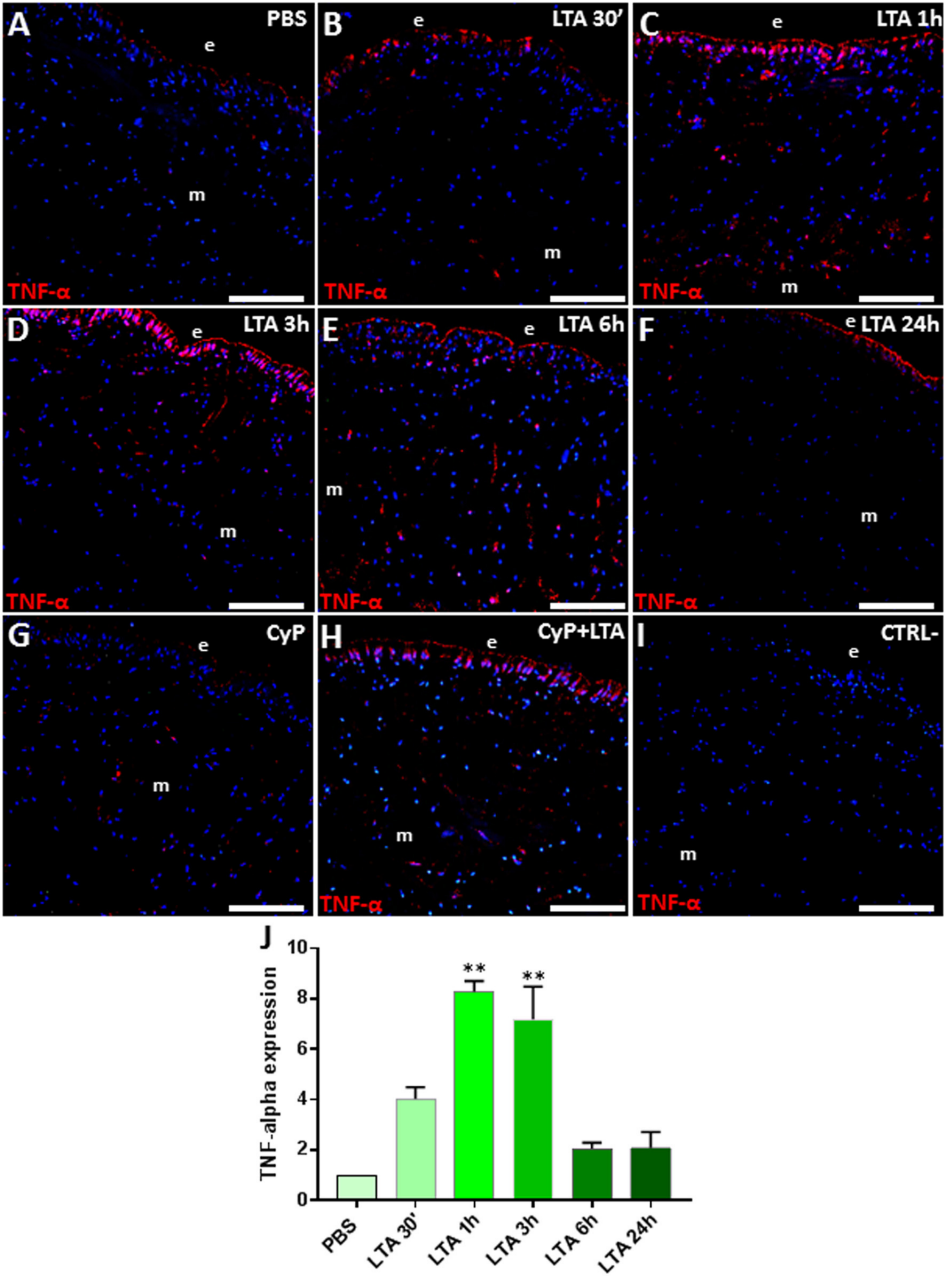
Immunofluorescence analyses on cryosection from leeches injected with PBS **(A)**, LTA **(B–F)**, CyP **(G)**, and CyP+LTA **(H)**. In PBS**-**injected leech **(A)**, few TNF-α cells were visible, whereas an increasing number of immune-responsive cells located underneath the epithelium (e) and in the ECM surrounding muscles (m) are detected starting from 30 min to 3 h following LTA treatment **(B–D)** and then decreases at 6 and 24 h **(E,F)**. In CyP **(G)** and Cyp/LTA samples **(H)**, the number of TNF-α^+^ cells is similar to that of PBS and of LTA-challenged leeches, respectively. In negative control experiments **(I)**, no positive cells are detected. The graph, relative to the Western blot analysis (see Figure S1), shows the TNF-α expression profile **(J)**. Bars in **(A–I)**: 100 μm. **p < 0.001.

Since one of the key targets of TLR2 signaling pathway is TNF-α (46), we also evaluated its expression level in control and LTA-stimulated leeches. Immunofluorescent assays detected a basal TNF-α expression in PBS-treated leeches (Figure 4A), whereas, as expected, its level markedly increased 30 min following LTA injection (Figure 4B), reached a peak at 1 and 3 h (Figures 4C,D), and then decreased at 6 h post-injection (Figures 4E,F). TNF-α expression in CyP- and CyP/LTA-injected leeches was comparable to that observed for TLR2 (Figures 4G,H), further confirming that the immune response caused by LTA treatment is not affected by blocking the TLR4 pathway. No signal was detected in the negative control experiments (Figure 4I). Western blot analysis of PBS- and LTA-injected leech tissues revealed the presence of an immunoreactive 36-kDa band (Figure S1), corresponding to the expected molecular mass of TNF-α in leech (30). A quantitative analysis confirmed the expression profile of this pro-inflammatory cytokine as already observed in immunofluorescence experiments (Figure 4J).

Taken together, these data showed that LTA injection in leeches triggered the expected innate immune response molecular signaling cascade, thus properly mimicking the effects of a real bacterial infection.

## *Hv*RNASET2 Expression in Immune-Competent Cells

**Figure 5:**
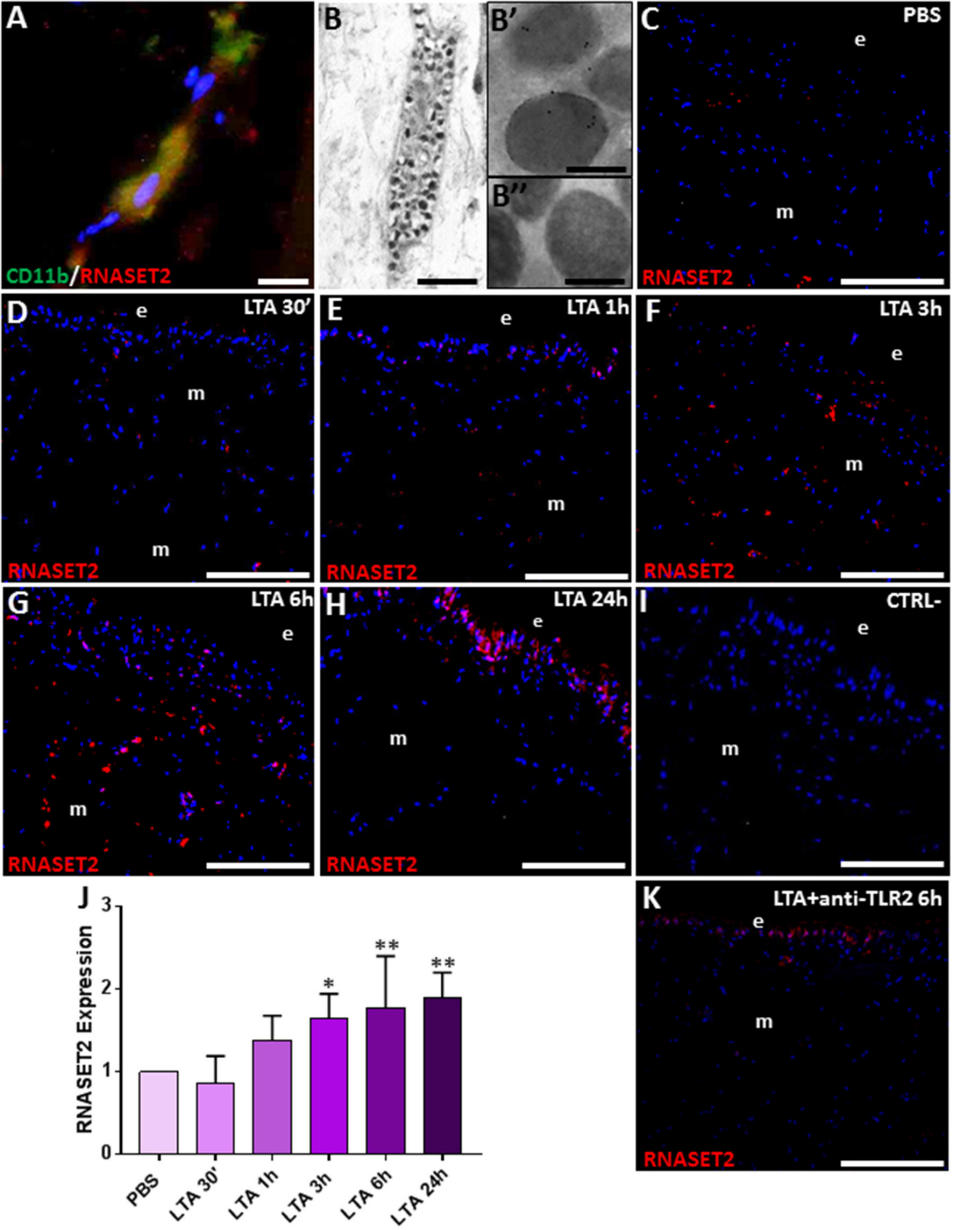
Double immunolocalization of RNASET2 (in red) and CD11b (in green) **(A)**. Immunogold staining shows that HvRNASET2 is localized in the electrodense granules **(B,B′)** of granulocytes **(B)**. No gold particles are detected in the negative control experiments, in which primary antibody is omitted **(B″)**. Immunofluorescence analyses on cryosection from leeches injected with PBS **(C)** and LTA **(D–H)** reveals an increasing RNASET2 signal starting from 30 min post LTA treatment. Negative control **(I)**. The graph, relative to the Western blot analysis (see Figure S1), shows the *Hv*RNASET2 expression profile **(J)**. After 6 h from injection of the antibody against TLR2, followed by LTA treatment, a low RNASET2 signal is found underneath the epithelium (e) and among muscles (m) **(K)**. Bar in **(A)**: 10 μm; bar in **(B)**: 2 μm; bars in **(B′,B″)**: 200 nm; bars in **(C–I,K)**: 100 μm. *p < 0.05, **p < 0.001.

We next carried out double immunofluorescence assays with anti-RNASET2 and anti-CD11b antibodies, coupled to immunogold experiments at TEM, in order to show the presence of *Hv*RNASET2 in the granules of leech granulocytes (Figures 5A–B″), as previously reported in LPS-stimulated leeches (17). In addition, *Hv*RNASET2 immunolocalization on cryosections from control and LTA-injected leech body wall showed that, in control, PBS-injected animals, *Hv*RNASET2 was expressed at a basal level (Figure 5C), whereas 30 min after LTA treatment, its expression gradually increased, reaching a peak after 6 and 24 h from stimulation (Figures 5D–H). No signal was visible when sections were incubated with the secondary antibody only (Figure 5I).

The *Hv*RNASET2 temporal expression profile in LTA-injected leeches was also evaluated by Western blot analyses. A 37-kDa immunoreactive band, corresponding to the molecular weight of the extracellular RNASET2 isoform, was detected in all samples (Figure S1). As expected, unlike control samples, the expression level of *Hv*RNASET2 gradually increased in LTA-treated samples, reaching a peak at 6 h post-treatment (Figure 5J).

To further confirm that the increased expression of *Hv*RNASET2 was TLR2-LTA mediated, we included a control reaction by injecting an anti-TLR2 antibody before LTA challenge. Immunofluorescence analysis showed that, after 6 h from LTA injection, the anti-TLR2 antibody treatment significantly reduced the RNASET2-induced response (Figure 5K) above described.

Taken together, our results not only confirmed that *Hv*RNASET2 was produced by leech granulocytes but also suggested that LTA-induced increase of this protein might be functionally involved in its potential antimicrobial activity against Gram-positive bacteria.

### Immune and Enzyme Histochemical Characterization of LTA-Recruited Macrophages

**Figure 6:**
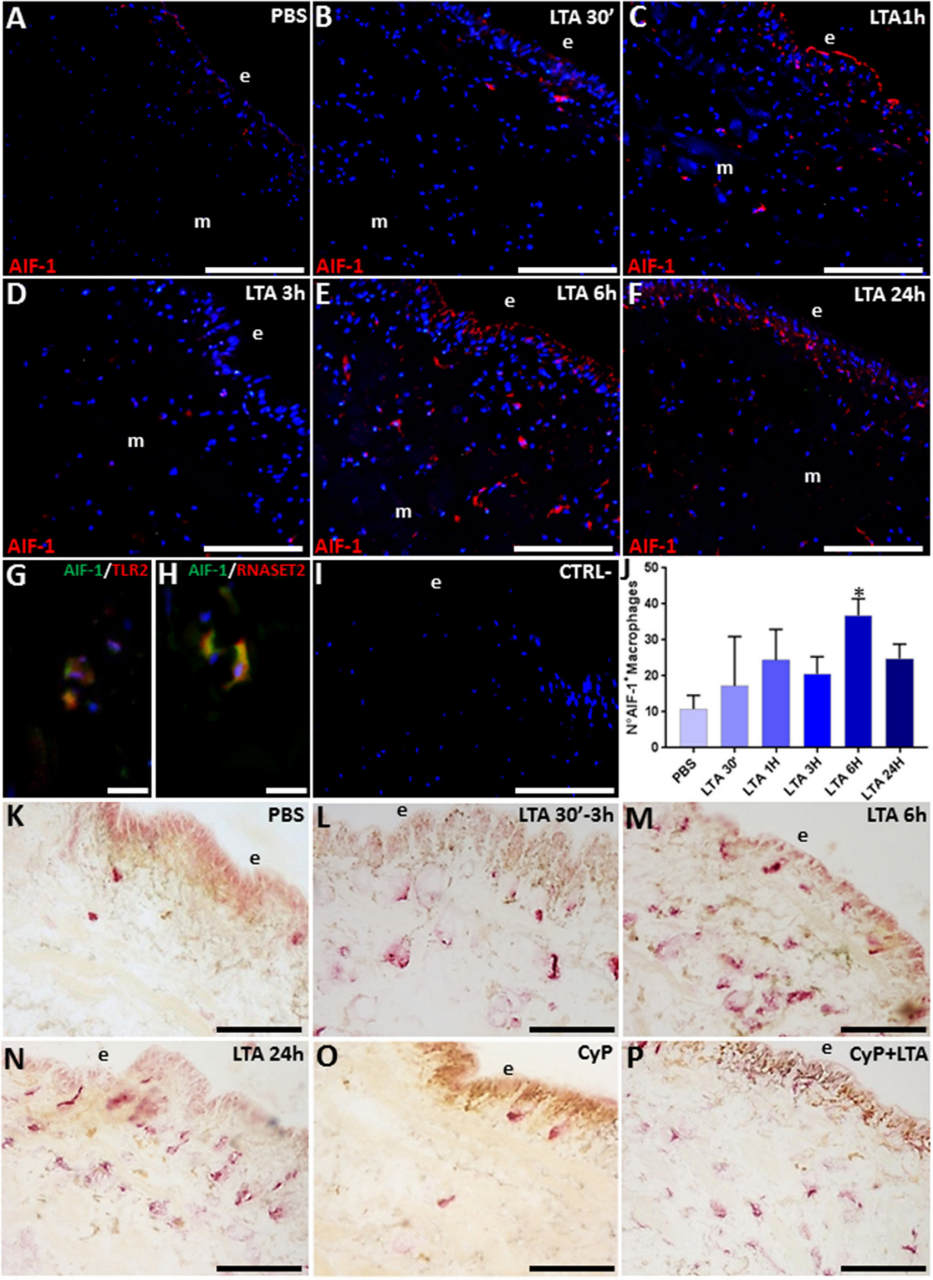
Immunofluorescence images of cryosection from leeches injected with PBS **(A)** and LTA **(B–F)**. In PBS-injected leech **(A)**, few *Hm*AIF-1^+^ macrophages are visible, while starting from 30 min up to 24 h from LTA injection, an increasing number of positive cells were detectable underneath the epithelium (e) and among the muscle fibers **(B–F)**. Double immunofluorescent assays **(G,H)**, using anti-*Hm*AIF-1 (in green) coupled to anti-TLR2 (in red) or anti-RNASET2 (in red) antibodies, reveal that macrophages (in yellow) are *Hm*AIF-1^+^/TLR2^+^ and *Hm*AIF-1^+^/RNASET2^+^. Negative control experiment **(I)**. The cell count is obtained on *Hm*AIF-1 fluorescence signal for each treatment **(J)**. The acid phosphate (ACP) reaction **(K–P)** shows an increasing cytosolic lysosomal activity in phagocytic macrophages after LTA challenging **(L–N)**. In CyP **(O)** and Cyp/LTA samples **(P)**, the number of ACP^+^ cells is similar to that of PBS- and of LTA-challenged leeches, respectively. Bars in **(A–F,I,K–P)**: 100 μm; bars in **(G,H)**: 10 μm. *p < 0.05.

The massive migration of leech granulocytes into the injected area during the earliest phase of the innate immune response was followed by a later macrophage recruitment toward the same area. Indeed, immunofluorescence analysis using the specific *Hm*AIF-1 macrophage marker unveiled the presence of many *Hm*AIF-1^+^ macrophages in the LTA-challenged area, while in control, PBS-injected leeches, only few resident cells were detectable next to the epithelium and in the ECM surrounding the muscle fibers (Figures 6A–F).

Strikingly, the co-localization pattern observed by double immunofluorescence assays with anti-*Hm*AIF-1 and anti-TLR2 or anti-RNASET2 antibodies suggested a direct involvement of the recruited macrophages in LTA response through the TLR2 pathway and confirmed that these immunocompetent cells expressed *Hv*RNASET2 as well during LTA-induced innate immune response, as previously observed after LPS challenging (16, 17) (Figures 6G–I). Indeed, *Hm*AIF-1^+^ cell counts carried out on five representative images of each time lapse confirmed a significant migration of macrophages, especially at 6 and 24 h post-treatment (Figure 6J).

Moreover, the enzymatic histochemical ACP assay (Figures 6K–P) revealed that these macrophages were actively involved in phagocytosis (16, 47–49), since PBS-injected leeches displayed a negligible number of ACP^+^ cells (Figure 6K), whereas their number significantly increased in relation to the time elapsed after LTA treatment, reaching a peak at 6 and 24 h from LTA injection (Figures 6M,N). The fact that CyP treatment did not induce any migration of phagocytic cells (Figure 6O) and at the same time did not reduce the LTA effects (Figure 6P) prompted us to postulate that the phagocytic activity of macrophages against Gram-positive bacteria was mediated by TLR2.

## *In vitro and in vivo* Assays to Evaluate the r*Hv*RNASET2 Effect

**Figure 7:**
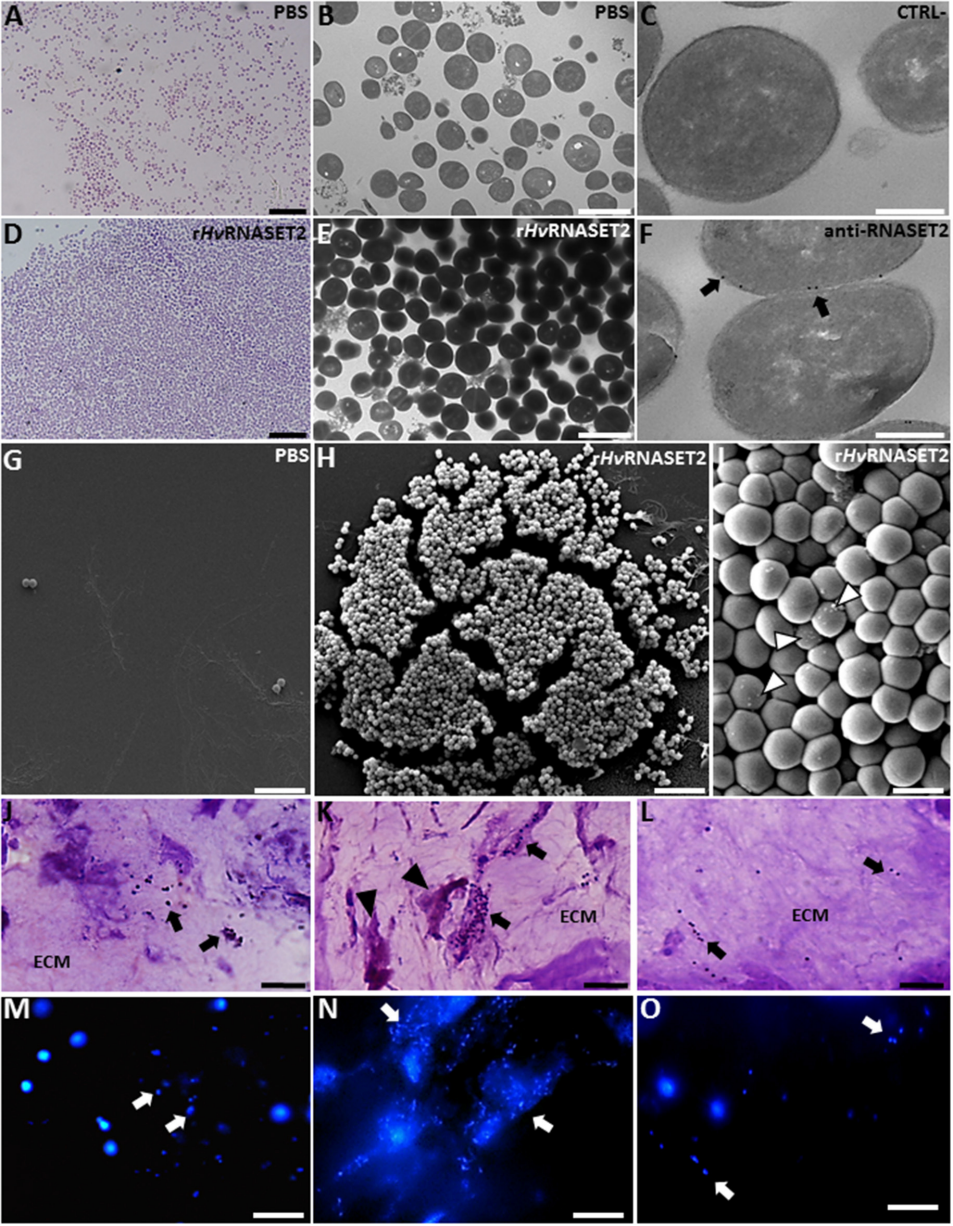
Evaluation of r*Hv*RNASET2 antibacterial activity by i*n vitro* **(A–I)** and *in vivo* **(J–O)** assays. Images at light **(A,D)**, TEM **(B,E)**, and SEM (**G–I**) microscopy of *S. aureus* incubated for 3 h *in vitro* with PBS or with r*Hv*RNASET2 show that only r*Hv*RNASET2 treatment induces a bacterial agglutination and the formation of several blebs on the bacterial cell surface (arrowheads in **I**). Immunogold assay **(C,F)** demonstrates a direct interaction between r*Hv*RNASET2 and the bacterial cell wall. *In vivo* experiments, performed by injecting in the leech body wall *S. aureus* alone **(J,M)** or *S. aureus* together with r*Hv*RNASET2 **(K,N)** or with r*Hv*RNASET2 pre-incubated with a specific blocking antibody **(L,O)**. The agglutination effect of r*Hv*RNASET2 on *S. aureus* is highlighted with violet and fuchsin colorant **(J–L)** and with fluorescent DAPI staining **(M–O)**. Bars in **(A,D,G,H,J–O)**: 10 μm; bars in **(B,E)**: 2 μm; bars in **(C,F)**: 250 nm; bar in **(I)**: 1 μm.

To further shed light on the putative *Hv*RNASET2-mediated antimicrobial role, we carried out morphological analyses by means of optical, TEM, and SEM microscopy on bacterial cell cultures upon incubation with 10 μM r*Hv*RNASET2 (Figures 7A–I).

Unlike control, PBS-treated samples (Figures 7A,B,G), adding *Hv*RNASET2 clearly induced the formation of bacterial clumps *in vitro* (Figures 7D,E,H,I). Strikingly, immunogold TEM analysis, using an anti-RNASET2 antibody, showed several gold particles localized on the bacterial cell surface, indicating a direct interaction between *Hv*RNASET2 and one or more bacterial cell wall components (Figures 7C,F). Moreover, several blebs could be observed by SEM analysis following *Hv*RNASET2 treatment, suggesting a local destabilization taking place at the bacterial cell surface (Figure 7I). However, r*Hv*RNASET2 *in vitro* did not apparently impair *S. aureus* viability on its own.

Of note, r*Hv*RNASET2 protein also triggered bacterial aggregation in *in vivo* experiments (Figures 7J–O). Indeed, both light and fluorescence images showed that, in leeches injected with either *S. aureus* alone or a mixture of the same bacterial cells and r*Hv*RNASET2 protein pre-incubated with a neutralizing anti-RNASET2 antibody, bacterial cells appeared randomly distributed throughout the leech ECM. By contrast, the extracellular matrix of leech co-injected with *S. aureus* and r*Hv*RNASET2 was consistently characterized by clusters of aggregated bacteria surrounded by macrophages (Figures 7K,N).

### Evaluation of the Enhanced Macrophage Phagocytic Activity Mediated by *Hv*RNASET2

**Figure 8:**
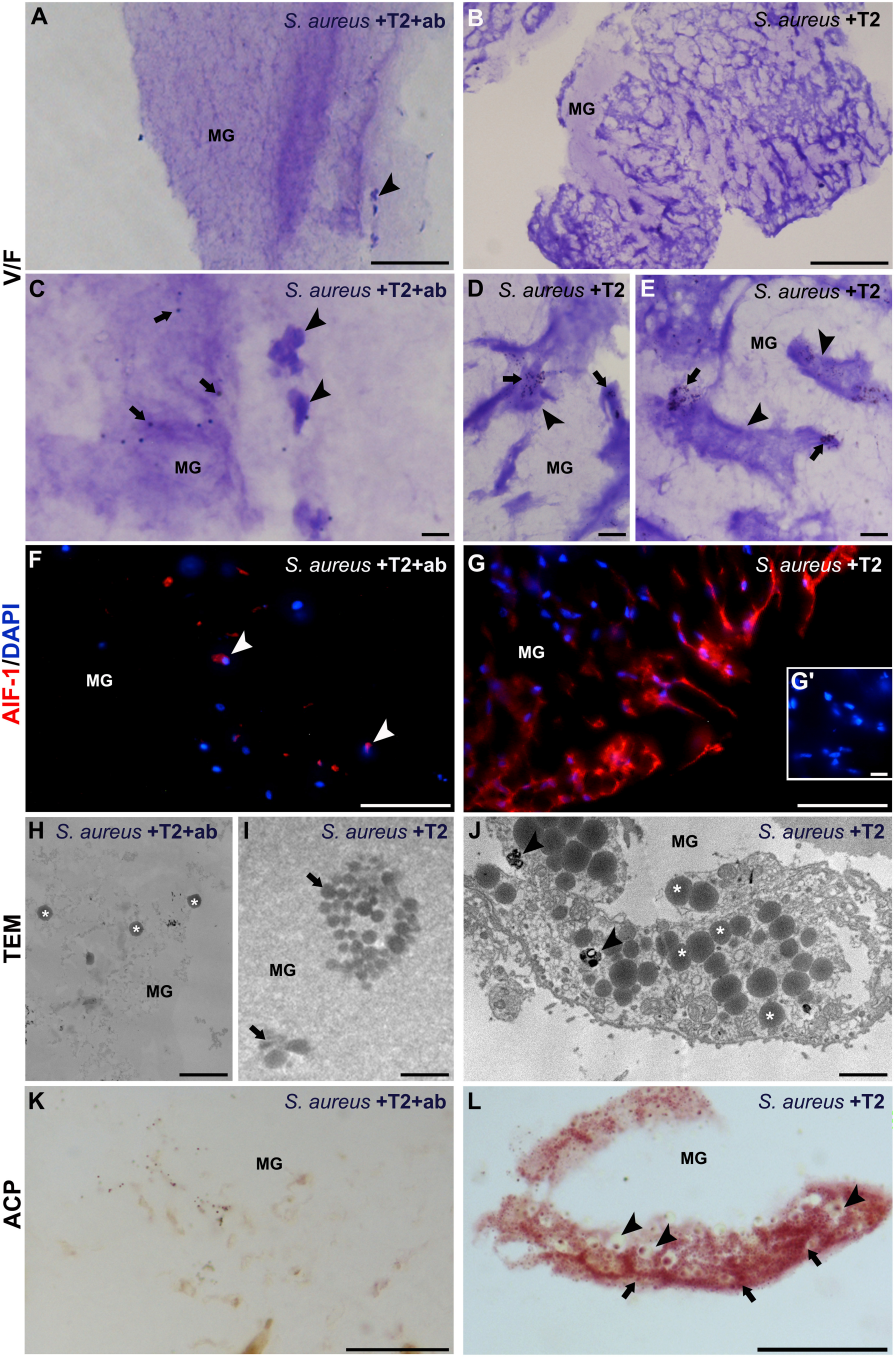
Morphological, immunofluorescence and histochemical analyses of matrigel (MG) sponges treated with *S. aureus*, r*Hv*RNASET2, and neutralizing antibody-pretreated RNASET2 **(A,C,F,H,K)**, used as control, or with *S. aureus* and r*Hv*RNASET2 **(B,D,E,G,I,J,L)**. After 1 week, randomly dispersed bacterial cells (arrows in **C**) and a few *Hm*AIF-1^+^ macrophages (arrowheads in **A,C,F**) are visible in control MG sponge. Contrariwise, MG pellet supplemented with *S. aureus* and r*Hv*RNASET2 is highly infiltrated by irregularly shaped macrophages, characterized by numerous pseudopodia (arrowheads in **B,D,E**) endowed in bacterial clumps phagocytosis (arrows in **D,E**) and highly expressing *Hm*AIF-1 **(G)**. Cell nuclei are stained in blue with DAPI. No signal is detected in negative control experiments in which the primary antibodies is omitted **(G′)**. **(H–J)** TEM images showing bacterial cells (asterisks in **H**) randomly distributed in control MG or forming clusters (arrow in **I**) in MG pellet supplemented with *S. aureus* and r*Hv*RNASET2 **(I)**. **(J)** Detail of a macrophage endowed with clustered *S. aureus* phagocytosis: numerous bacterial cells (asterisks) and phagolysosomes (arrowheads) are clearly visible in the cell cytoplasm. **(K,L)** A strong ACP reaction is detectable only in MG pellet supplemented with *S. aureus* and r*Hv*RNASET2 **(L)** where phagocytic macrophages, showing phagolysosomes (arrowheads) and lysosomal activity (arrows) in their cytoplasm, are visible. Bars in **(A,B)**: 100 μm; bars in **(C)**: 20 μm; bars in **(D,E,G′,L)**: 10 μm; bars in **(F,G,K)**: 50 μm; bars in **(H–J)**: 2 μm. *p < 0.05.

To further confirm the ability of r*Hv*RNASET2 in stimulating a macrophage-mediated phagocytic activity, *S. aureus* cells were pre-treated with the recombinant r*Hv*RNASET2 protein, added to MG biopolymer held at a liquid state and subsequently inoculated in leech body wall. The formed MG solid pellets were then explanted after 1 week and analyzed by morphological, immunofluorescence, and histochemical assays (Figures 8A–L). In control MG pellet (supplemented with *S. aureus* and r*Hv*RNASET2 pre-incubated with anti-RNASET2 antibody) (Figures 8A,C,F,H,K), bacterial cells appeared randomly distributed and a few infiltrating *Hm*AIF-1^+^ macrophages were detectable. These cells displayed a weak phagocytic activity as demonstrated by ACP low positivity. In striking contrast, MG samples containing r*Hv*RNASET2 and *S. aureus* showed several macrophages (Figures 8B,D,E) expressing *Hm*AIF-1^+^ (Figures 8G,G′). TEM analysis (Figures 8H–J) and ACP assay (Figures 8K,L) confirmed that only in MG samples containing r*Hv*RNASET2 and *S. aureus* were bacterial cells aggregated in clusters and macrophages endowed with high phagocytic activity (16).

This finding is clearly consistent with a role for r*Hv*RNASET2 in stimulating a macrophage-mediated antimicrobial response, likely carried out by bacterial aggregation followed by ingestion by r*Hv*RNASET2-recruited macrophages.

## Discussion

Although, in the last decades, several studies have demonstrated that members of the human RNase A superfamily play a crucial role in the defense against bacterial infection (50), very little is known about the potential antibacterial activity of T2 RNase family members and their possible involvement in microbial recognition.

We have previously demonstrated that the leech ribonuclease *Hv*RNASET2 not only acts as a chemoattractant for granulocytes and macrophages, being thus involved in modulating inflammatory processes, but also plays an effective response against Gram-negative bacteria infection (16, 17). Indeed, our previous data clearly demonstrated that LPS bacterial injection in the leech body wall induced an increased expression of endogenous *Hv*RNASET2 in both granulocytes and macrophages. Interestingly, *Hv*RNASET2 was released by granulocytes in the early phase of the inflammatory response, likely playing an antibacterial role against Gram-negative bacteria, by recruiting macrophages in the challenged area (16). Both macrophages and granulocytes are able to recognize Gram-negative bacteria by expressing on their membranes, the specific LPS receptor TLR4 (30). Recombinant RNASET2 did not apparently influence cellular viability *in vitro*, but it nevertheless affected *Pseudomonas aeruginosa* cell wall, triggering a change in the typical rod morphology of these cells (17).

Here, we have analyzed the antibacterial activity of *Hv*RNASET2 against Gram-positive bacteria, by investigating the role of this ribonuclease in regulating and orchestrating the innate immune response induced by stimulation with LTA in the medicinal leech *H. verbana*. This invertebrate has been increasingly used as an experimental model to study innate immune response processes, due to its cost-effective use, easy manipulation, and lack of significant ethical considerations related to regulatory restrictions, coupled to the occurrence of immune response processes that are very similar to those reported in vertebrates (15, 51–53).

By means of morphological, ultrastructural, and immunofluorescence analyses, we have first shown that LTA injection in the leech body wall induces a marked recruitment of CD11b^+^ granulocytes, which are the first immune cells to be activated following leech bacterial infection (17, 30). These immune cells were shown to express TLR2, the key cell receptor involved in the response to Gram-positive bacteria. Moreover, as in vertebrates, TLR2 triggering induced intracellular signaling events involving MyD88, a key molecular intermediate for the activation of TLR2 signaling pathways (54) that ultimately leads to the production of the proinflammatory cytokines such as TNF-α (29, 30, 55). Functional studies carried out by injecting the cyanobacterium selective TLR4 antagonist CyP strongly suggested that, as in vertebrates, the recognition of LTA (a component of Gram-positive cell wall) in leeches did not involve the TLR4 pathway, but was specific for the TLR2 pathway (54).

Interestingly, after 3 h from stimulation with LTA, CD11B^+^/TLR2^+^ granulocytes also expressed a high amount of *Hv*RNASET2, as demonstrated by double immunofluorescence and Western blot assays. Indeed, the functional blocking experiment, performed by injecting the anti-TLR2 antibody in the leech body wall followed by LTA treatment, confirms a close correlation between *Hv*RNASET2 expression and an LTA/TLR2-induced response.

This ribonuclease, as previously demonstrated following Gram-negative bacterial challenge, promoted macrophage migration and activation, since cells expressing the specific leech macrophage *Hm*AIF-1 (47, 56) were clearly recruited in the challenged area and showed a high phagocytic activity, as demonstrated by their positivity for the hystoenzymatic ACP reaction. We propose that such cells have a dual role following bacterial infection in leeches, being involved in both cleaning up the infected area from bacteria and producing, 24 h following LTA stimulation, a second wave of *Hv*RNASET2 for further macrophage recruitment. Of note, the phagocytic activity of macrophages was apparently facilitated by the aggregating properties of *Hv*RNASET2 on bacterial cells. Indeed, *in vitro* experiments clearly show that, following *Hv*RNASET2 treatment, *S. aureus* formed cell clusters. This aggregation was likely mediated by a specific interaction between *Hv*RNASET2 and the bacterial cell wall, as demonstrated by immunogold experiments at TEM. Thus, this aggregating activity is apparently not involved in direct killing of bacteria, as no change in *S. aureus* viability was observed when *Hv*RNASET2 was administered at increasing concentrations. This is in accordance with our previous findings where *Pseudomonas aeruginosa* expressing green fluorescent protein (GFP) were co-cultured with RNASET2-expressing THP-1 cells. Under the tested conditions, a GFP release pattern compatible with modified bacterial membrane permeability was observed. Although this change did not influence the cellular viability *in vitro*, we hypothesized that *in vivo* it could represent the first part of a multi-step antimicrobial activity involving other immunity cells (17).

Of note, the aggregating ability of *Hv*RNASET2 was also demonstrated by *in vivo* experiments, since clusters of *S. aureus* cells surrounded by macrophages were detected in the injected leech body wall in the presence of *Hv*RNASET2. Moreover, as visible *in vivo* MG experiments, the phagocytic macrophage activity turned out to be facilitated by bacterial cluster formation.

Taken together, our results clearly suggest for the first time that the antimicrobial properties of leech recombinant *Hv*RNASET2 protein are clearly comparable to those expressed by other class A RNases, such as RNase 3 and 7, and correlate to bacterial clumps forming activities (57). Indeed, similarly to RNase 7, *Hv*RNASET2 triggered local blebs in the bacterial wall (58) and promoted a marked inflammatory response, mediated by TLR2 activation and followed by the release of inflammatory stimuli, such as TNF-α (26). Moreover, as reported for RNase 3, the ability of *Hv*RNASET2 to bind bacterial cell wall seems of special relevance for its role in bacterial clumps formation (58).

To our knowledge, this study represents the first report describing a marked antibacterial activity for a T2 RNase member in an invertebrate experimental model, coupled with a plausible mechanism of action. Of note, the similarity of the biological role between *Hv*RNASET2 and some class A RNases opens the door to the production of new peptide-derived antimicrobial drugs, which could help counteract the ever-growing problem of the dramatic worldwide emergence of antibiotic-resistant strains.

## Data Availability Statement

All datasets generated for this study are included in the article/Supplementary Material.

## Author Contributions

AG and FA conceived and designed the experiments and wrote the main manuscript text. NB performed the morphological, immunofluorescence, immunogold, western blot analyses, and prepared all the figures. LM and AD performed statistical analysis. VO performed bacterial viability test and critically read the manuscript. MR provided expertise for SEM and imaging. ME and GT provided expertise for TEM and imaging. LP and ER contributed to reagents. All authors critically reviewed the manuscript.

### Conflict of Interest

The authors declare that the research was conducted in the absence of any commercial or financial relationships that could be construed as a potential conflict of interest.

## Supplementary Material

Figure S1 Western blot analysis performed on PBS or LTA injected leeches. Proteins extracted from leech body wall are probed with anti-TLR2 **(A)**, anti- TNF-α **(B)**, and anti-RNASET2 **(C)** antibodies, respectively. Each experiment is quantified on the expression level of D-glyceraldyde-3-phosphate dehydrogenase (GAPDH), used as a control. Immunoreactive bands of about 109, 34, and 37 kDa are, respectively detected for anti-TLR2, anti-TNF-α, and anti-RNASET2.
