# Supplementary figures and images for: Antimicrobial Role of RNASET2 Protein During Innate Immune Response in the Medicinal Leech Hirudo verbana
Source: Front Immunol. 2020 Mar 6;11:370. doi: 10.3389/fimmu.2020.00370 (PMC7068815; doi:10.3389/fimmu.2020.00370)

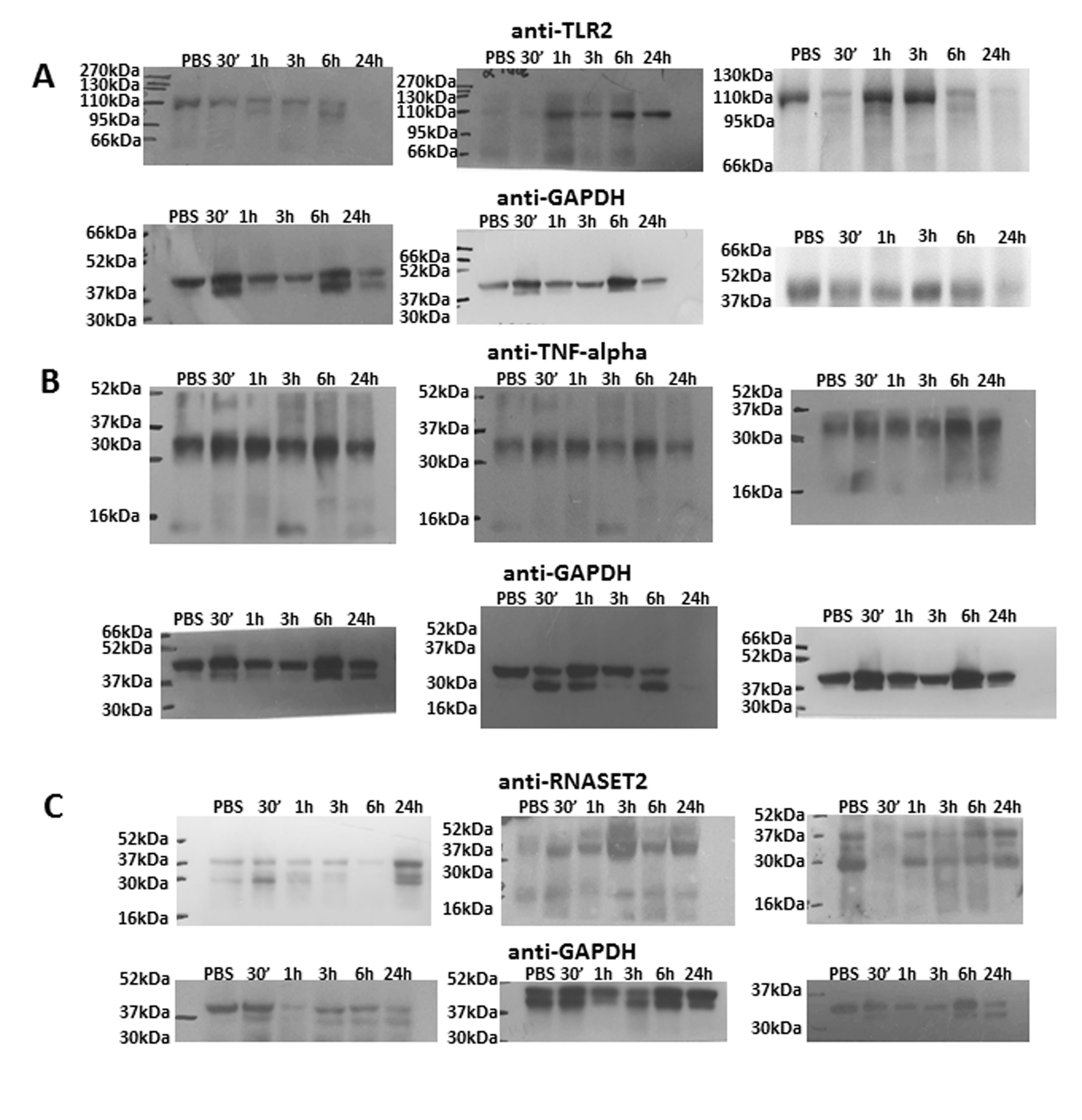

Supplement: Figure S1 — Western blot analysis performed on PBS or LTA injected leeches. Proteins extracted from leech body wall are probed with anti-TLR2 (A), anti- TNF-α (B), and anti-RNASET2 (C) antibodies, respectively. Each experiment is quantified on the expression level of D-glyceraldyde-3-phosphate dehydrogenase (GAPDH), used as a control. Immunoreactive bands of about 109, 34, and 37 kDa are, respectively detected for anti-TLR2, anti-TNF-α, and anti-RNASET2. [file Image_1.TIF]
